# Appendectomy for suspected appendicitis during pregnancy– a retrospective comparative study of 99 pregnant and 1796 non-pregnant women

**DOI:** 10.1007/s00423-024-03517-3

**Published:** 2024-10-28

**Authors:** Michael Hoffmann, L. Anthuber, A. Herebia da Silva, A. Mair, S. Wolf, C. Dannecker, M. Anthuber, M. Schrempf

**Affiliations:** 1 Department of General, Visceral and Transplantation Surgery, University Hospital Augsburg, Stenglinstrasse 2, 86156 Augsburg, Germany; 2 Department of General, Visceral and Minimally-Invasive Surgery, City Hospital Bad Toelz, Schützenstrasse 15, 83646 Bad Toelz, Germany; 3 Department of Gynecology and Obstetrics, University Hospital Augsburg, Stenglinstrasse 2, 86156 Augsburg, Germany

**Keywords:** Appendicitis, Pregnancy, Emergency surgery, Negative appendectomy

## Abstract

**Introduction:**

Suspected appendicitis is the most common indication for non-obstetric surgery during pregnancy. Diagnosis and management of these patients can be challenging. Atypical clinical presentation has been described before, but the current literature consists mostly of small case series. Therefore, we conducted a large retrospective study to analyze the frequency and diagnostic accuracy of clinical signs, laboratory findings and imaging modalities in pregnant woman undergoing surgery for suspected appendicitis compared to a control group of non-pregnant women of childbearing age. We further describe intra- and postoperative findings in both groups.

**Methods:**

Data from consecutive patients who underwent appendectomy for suspected appendicitis during pregnancy were retrieved from the electronic patient database and analyzed. Preoperative clinical, laboratory and imaging findings as well as intra- and postoperative characteristics were compared between pregnant and non-pregnant women.

**Results:**

Between January 2008 and June 2023, 99 pregnant woman and 1796 non-pregnant woman between the ages of 16 and 49 underwent emergency surgery for suspected appendicitis. Pregnant women were less likely to have right lower quadrant tenderness (*p* = 0.002), guarding (*p* = 0.011) and rebound tenderness (*p* = 0.097). A greater percentage of pregnant women had a symptom duration of more than 24 h before presentation (*p* = 0.003) Abdominal ultrasound showed a reduced diagnostic accuracy in pregnant women (*p* = 0.004). MRI was used in eight pregnant women and showed a diagnostic accuracy of 100%. Pregnant women had a longer operating time (*p* = 0.006), a higher rate of open appendectomies or conversion (*p* < 0.001) and a longer postoperative hospital stay (3.2 days vs. 2.2 days, *p* < 0.001). The perforation rate was also higher in pregnant women at 16% vs. 10% (*p* = 0.048).

**Conclusion:**

The diagnosis of acute appendicitis during pregnancy presents a challenge for the clinician. Our data confirm the paradigm of “atypical presentation” which should lead to an extended diagnostic workup. Ultrasound showed less diagnostic accuracy in pregnant women in our study. MRI is a useful tool to reduce uncertainty and the rate of negative appendectomies.

## Introduction

With an incidence ranging from 1:500 to 1:1000 per pregnancy, suspected appendicitis is the most common indication for non-obstetric surgery during pregnancy [1, 2]. Management of these patients can be challenging for several reasons.

First, correct and timely diagnosis is of great importance as perforation can affect maternal and neonatal health [3, 4]. However, negative appendectomy during pregnancy is also associated with adverse neonatal outcome [5, 6]. Clinical, laboratory and radiological findings are used for diagnosis. Pregnant women are less likely to have a classical clinical presentation of acute appendicitis [3]. Gastrointestinal symptoms and abdominal discomfort are common during pregnancy. In addition, the localization of pain may be atypical due to anatomical changes. As leukocytosis may be a normal finding in pregnant women, Laboratory tests such as white blood cell count (WBC) and C-reactive protein (CRP) may also be of limited value, as mild leukocytosis including a slight left shift is frequently found during pregnancy [3].

Routine imaging is recommended in international guidelines [7]. The *American College of Radiology (ACR) Appropriateness Criteria* for pregnant women recommend abdominal ultrasound as first method of choice for suspected appendicitis [8]. The main limitation of ultrasound is non-visualization of the appendix, especially beyond the first trimester. Computed tomography (CT) should be avoided due radiation exposure. Magnetic resonance imaging (MRI) has a high diagnostic accuracy and should be used in cases of inconclusive ultrasound findings [9, 10]. Alternatively, it can be used as first imaging modality [11]. However, availability is limited in many places, especially at night. Since a negative or inconclusive MRI does not completely rule out appendicitis, surgery should still be considered in cases of high suspicion [7].

Non-operative management of uncomplicated acute appendicitis is not recommended during pregnancy due to limited evidence. Furthermore, according to a recent study, it is associated with worse clinical outcome compared to appendectomy [12].

There has been an ongoing debate about the role of laparoscopy during pregnancy, but meta-analyses have shown its safety in terms of risk of fetal loss and preterm delivery [13]. Therefore, laparoscopic appendectomy during pregnancy should be preferred over open surgery when feasible in terms of surgeon experience and equipment availability [7].

Despite the frequency of appendicitis during pregnancy, the current literature consists mostly of small case series. The aim of this study was to analyze the frequency and diagnostic accuracy of typical clinical signs, laboratory findings and imaging modalities in pregnant woman undergoing surgery for suspected appendicitis compared to a control group of non-pregnant women of childbearing age. Furthermore, we describe intra- and postoperative findings for both groups. This is one of the largest retrospective studies from a single institution and will add to the body of knowledge on the surgical management of appendicitis during pregnancy.

## Methods

This study was conducted at the Department of General, Visceral and Transplant Surgery at the University Hospital Augsburg, Germany, as a single center retrospective study. Our department provides a tertiary center healthcare to the entire metropolitan region of Augsburg and surrounding area. The study was approved by the Ethics Committee of the Ludwig Maximilians University (LMU), Munich (reference number 23–0500) and conducted in accordance with the Declaration of Helsinki.

### Study population, definitions and surgical procedure

We identified all women aged between 16 and 49 years who underwent emergency surgery for suspected appendicitis at our institution between January 2008 and June 2023 from the institutional electronic database. Within this cohort of 1895 women we identified those who were pregnant at the time of surgery. This gave a cohort of 99 pregnant women. The cohort of non-pregnant women of childbearing age (*n* = 1796) served as control group. No patients were excluded from this patient population.

The diagnosis was made by the attending surgeon after a clinical examination, laboratory values and sonography were performed, and an obstetric cause of the symptoms was ruled out by a gynecologist. An MRI was performed at the discretion of the surgeon on duty and when available. All patients were examined by a gynecologist and a surgeon before undergoing surgery. Negative appendectomy was defined as uninflamed appendix following the final histopathological result.

The choice of surgical technique was made by the surgeon on duty. Laparoscopic appendectomy was the standard procedure in non-pregnant women as well as in the first and second trimester. The primary trocar was placed above the umbilicus via mini-laparotomy. Veress needle was not used in pregnant women. To facilitate the operation, Trendelenburg position was used with a left lateral inclination, usually 10 to 15 degrees towards the surgeon. The appendix was grasped with an atraumatic forceps. Blunt atraumatic forceps was also used for careful retraction of the uterus, if necessary.

Electronic health records were reviewed, and perioperative data were extracted. Complications, comorbidities, operative data and patient characteristics were collected from the database, including age, ASA status, BMI, preoperative symptoms, preoperative CRP leukocyte and bilirubin levels, radiologic findings, time to surgery, intraoperative findings, operating time, percentage of laparoscopic procedures, histopathology results, complication rate and length of hospital stay. Complications were assessed using the Clavien-Dindo classification and the Comprehensive Complication Index^®^ (CCI) [14, 15].

### Statistical analysis

Continuous data is presented as mean ± standard deviation or median with interquartile range, depending on distribution. Categorical data is presented as numbers with percentages. Continuous variables were compared using the independent t-test and the Mann-Whitney-U test depending on distribution. Categorical data was compared using the χ2 test. Fisher’s exact test was used for categorical data when the requirements for χ2 test were not met. A two-sided *P* < 0.05 was considered significant. Demographic, clinical, laboratory and radiological findings were tested for differences between both cohorts in a univariate analysis. Furthermore, intra- and postoperative characteristics such as histopathologic findings and complication rates were compared between the two groups. Statistical analyses were undertaken using SPSS^®^ for macOS^®^, version 31 (IBM, Armonk, New York, USA).

## Results

We identified 1895 women between 16 and 49 years who underwent emergency surgery for suspected appendicitis at our institution between January 2008 and June 2023. 99 of these women were pregnant. The 1796 non-pregnant women served as control group. Of the 99 pregnant women, 22 were in the first trimester, 47 in the second trimester and 30 in the third trimester.

### Preoperative factors

The group of pregnant women had a mean age of 30.1 years compared to 28 years in the cohort of non-pregnant women. The demographic characteristics and preoperative findings are shown in Table 1. Clinical presentation showed some differences between the two groups. While the frequency of pain migration and nausea or vomiting was similar, pregnant women were less likely to have right lower quadrant tenderness (*p* = 0.002) and guarding (*p* = 0.011). The frequency of rebound tenderness showed a trend towards lower rates in the pregnant group (*p* = 0.097). In 48.1% of pregnant women compared to 31.8% of non-pregnant women, symptoms were already present more than 24 h before presentation (*p* = 0.003). CRP levels on admission did not differ between groups, but pregnant women showed higher WBC levels (*p* = 0.014). We found a strong association between leukocytes above 18 per nl and perforation in our cohort of pregnant women (*p* = 0.002; OR 5,68; 95% KI 1,73 − 18,63). This association was also found for CRP levels above 10 mg/dl (*p* < 0.001; OR 11,98; 95% KI 3,14–45,71).

Abdominal ultrasound showed a sensitivity of 47.5% in pregnant women compared to 63.5% in non-pregnant women (*p* = 0.004). MRI was used in eight pregnant women (8.1%) compared to less than 1% in the control group. The diagnostic accuracy in these eight patients was 100%. The time from admission to surgery did not differ between the two groups.

**Table 1:** Demographic and preoperative characteristics

	Pregnant n = 99	Non pregnant n = 1796	p
Age	30.1 ± 5.8	28.0 ± 9.0	< 0.001
BMI*	27.5 ± 5.7	24.0 ± 5.2	< 0.001
RLQ Tenderness			0.002
- Yes	83 (83.8)	1659 (92.4)	
- No	16 (16.2)	137 (7.6)	
Pain migration			0.27
- Yes	17 (17.2)	392 (21.8)	
- No	82 (82.8)	1404 (78.2)	
Guarding			0.011
- Yes	23 (23.2)	642 (35.7)	
- No	76 (76.8)	1154 (64.3)	
Rebound Tenderness			0.097
- Yes	20 (20.2)	500 (27.8)	
- No	79 (79.8)	1296 (72.2)	
Nausea and / or vomiting	25 (25.3)	498 (27.7)	0.59
- Yes			
- No	74 (74.7)	1298 (72.3)	
Duration of symptoms prior to presentation at ED			0.003^‡^
- < 24h	60 (68.2)	893 (51.9) ^‡^	
- ≥ 24h	28 (31.8)	828 (48.1) ^‡^	
- unknown	11	75	
WBC (per nl)	13.6 ± 4.5	12.5 ± 4.5	0.014
CRP (mg/l)	40.2 ± 46.9	37.1 ± 55.6	0.30
Bilirubin (mg/dl)	0.48 ± 0.35	0.60 ± 0.43	0.009
Ultrasound (direct or indirect signs)			< 0.001
- Positive	41 (42.3)	1052 (60.1)	
- Negative	56 (57.7)	698 (39.9)	
No ultrasound available	2	46	
Ultrasound (direct signs)			0.003
- Positive	33 (34.0)	865 (49.4)	
- Negative	64 (66.0)	885 (50.6)	
No ultrasound available	2	46	
Perforation on Ultrasound			0.18
- Yes	1 (1.0)	69 (4.0)	
- No	96 (99)	1681 (96.0)	
No ultrasound available	2	46	
Ultrasound: sensitivity	47.5%	63.5%	0.004
Ultrasound: specifity	82.4%	55.8%	0.032
Admission to surgery			0.90
≤ 12 hours	71 (71.7)	1299 (72.3)	
> 12 hours	28 (28.3)	497 (27.7)	

Data are mean ± SD or n (%) or median (IQR)

IQR interquartile range, SD standard deviation, BMI body mass index, CRP C-reactive protein, WBC white blood cell

^‡^ Percentages and p-value refer to subgroup of patients with known duration of symptoms

* BMI was recorded at the time of hospital admission

### Intra- and postoperative characteristics

Intra- and postoperative findings are shown in Table 2. Pregnant women had a longer operating time (*p* = 0.006), a higher rate of open appendectomies or conversion (*p* < 0.001) and a longer postoperative hospital stay (3.2 days vs. 2.2 days, *p* < 0.001). The rate of laparoscopically performed appendectomies decreased from 99.5% (21/22) in the first trimester to 85.1% (40/47) in the second trimester and 33.3% (10/30) in the third trimester.

The rate of perforations was also higher in pregnant women with 16% vs. 10% (*p* = 0.06). They showed a higher complication rate measured by the Comprehensive Complication Index (CCI^®^), but most of them were mild. Two cesarean sections had to be performed within 30 days after appendectomy which were classified as complications Clavien-Dindo ≥ 3. One patient at a gestational age of 34 weeks presented with a pathological CTG one day after appendectomy. Cesarean section was performed immediately. The second patient at a gestational age of 24 weeks was readmitted 3 weeks after appendectomy. The cesarean section was performed because of suspected chorioamnionitis. Both patients had pathologically confirmed appendicitis. No other severe complication occurred among pregnant women. One additional patient suffered miscarriage at a gestational age of 9 weeks, one day after appendectomy for ulcero-phlegmonous appendicitis. This was not classified as maternal complication. The rate of negative appendectomies was 18% among pregnant women compared to 16.5% in non-pregnant women (*p* = 0.7).

**Table 2:** Intraoperative and postoperative characteristics

	Pregnant	Non pregnant	*p*	Mean difference
Operating time (min)	50 (IQR 40–62)	55 (IQR 43–71)	0.006 ^(1^	6.1
Length of postoperative stay (days)	3.2 (IQR 2.4–5.7)	2.2 (IQR 1.7–2.8)	< 0.001 ^(1^	1.4
CCI^®^ (range 0-100)	4.6 ± 9.0	0.71 ± 4.05	< 0.001 ^(1^	3.9
	Pregnant	Non pregnant	p	OR (95% CI)
Complication Clavien-Dindo ≥ 3	2 (2.0%)	15 (0.8%)	0.22 ^(2^	2.45 (0.55–10.86)
Open appendectomy or conversion to open appendectomy	28 (28.3%)	54 (3.0%)	< 0.001 ^(2^	12.72 (7.61–21.28)
Negative appendectomy*	15 (15.2%)	293 (16.3)	0.76 ^(2^	0.92 (0.52–1.61)
Complicated appendicitis	58 (58.6%)	970 (54.0%)	0.37 ^(2^	1.21 (0.80–1.82)
Perforation	16 (16.2%)	179 (10.0%)	0.06 ^(2^	1.74 (0.998–3.04)

Data are mean ± SD or n (%) or median (IQR)

IQR interquartile range, SD standard deviation, BMI body mass index, CRP C-reactive protein, WBC white blood cell

* Defined as no signs of inflammation or other pathologic finding on histologic examination

(1 Mann-Whitney-U-Test

(2 Chi-Squared-Test / Fishers’ exact test

## Discussion

The diagnosis of acute appendicitis during pregnancy can be challenging. To our knowledge, this is the largest study in the literature comparing clinical and diagnostic findings between pregnant and non-pregnant women.

In our study, the clinical signs differed between the two cohorts in some aspects. The “classic presentation” of acute appendicitis usually starts with abdominal pain which often begins in the periumbilical region and then migrates to the right lower quadrant as the inflammatory process progresses [16]. Anorexia, nausea and vomiting are typical symptoms, but of low discriminatory power. Right lower quadrant tenderness, guarding and rebound tenderness are common and have a higher predictive value [17]. It has been previously described that pregnant patients are less likely to have a classic presentation [3]. Especially in the second and third trimester, pain localization can be atypical [18]. In our study, we found a lower rate of right lower quadrant tenderness, guarding and rebound tenderness in pregnant patients. Not many studies address these signs and symptoms in depth, but a recent publication also found a lower rate of right lower quadrant tenderness in pregnant women [19]. Symptom duration of more than 24 h is usually associated with a lower likelihood of appendicitis [17]. In our study, far more pregnant than non-pregnant women had a symptom onset of more than 24 h prior to presentation at the emergency department. Our data confirm the paradigm of “atypical presentation” which in turn should lead to an extended diagnostic workup.

The WSES guidelines recommend that the diagnosis of acute appendicitis in pregnant patients should not be made based on symptoms and clinical signs alone. Laboratory tests and inflammatory serum parameters should always be obtained [7]. A higher rate of negative appendectomies during pregnancy has been considered acceptable in the past. However, unnecessary surgical procedures should be avoided, as they are associated with an unfavorable neonatal outcome [5, 6].

WBC and CRP are widely used as inflammatory parameters. Mild leukocytosis may be a normal finding in pregnant women [20] which makes the interpretation of this parameter difficult. In our study, pregnant women had a slightly higher WBC with a mean of 13.5 vs. 12.5 per nl. This is in contrast to the results of some other publications, which found no difference [4, 19, 21]. Some studies have shown that a WBC value over 18 per nl is highly predictive of appendicitis in pregnant women [22]. This was also true in our population, but there was no significant difference between the two groups with regard to positive predictive value (88.2% for pregnant vs. 93.2% for non-pregnant women). CRP levels were similar between both groups. Based on our findings it can be concluded that the value of inflammatory parameters does not differ between pregnant and non-pregnant women. Nevertheless, we could show that caution is warranted if leukocytes are above 18 per nl or CRP level is above 10 mg/dl because we found a strong association with perforation in the cohort of pregnant women.

In agreement with previous literature, ultrasound was the first imaging modality for suspected appendicitis at our institution [7, 23] and was performed in over 97% of pregnant and non-pregnant women. The attributable risk of cancer due to ionizing radiation for women in reproductive age is higher than previously thought [24] and should be avoided, especially during pregnancy, as both mother and fetus are exposed. We did not perform CT examinations in pregnant women. In our study, the diagnostic accuracy and sensitivity of ultrasound were significantly lower in pregnant patients, which is a challenge for the clinician. Eight pregnant women (8.1%) compared to less than 1% in the control group underwent MRI because of inconclusive ultrasound and unclear clinical findings. This number appears relatively low, which might be due to two reasons: (1) the time period of our study, dating back to 2008, when recommendations regarding MRI were less clear, (2) the availability during night time, which is not always given in our institution. The diagnostic accuracy in these eight patients was 100%, suggesting more frequent use in the future. A recent meta-analysis confirms a high sensitivity and specificity of over 90% for the diagnosis of acute appendicitis in pregnant women with clinically suspected appendicitis [25]. The authors conclude that MRI is an excellent option for pregnant patients with suspected acute appendicitis. In accordance with these findings, the WSES guidelines recommend MRI during pregnancy if the ultrasound examination is inconclusive [7]. In cases of equivocal diagnosis, observation and reassessment in short periods also reduce negative appendectomies without leading to more perforations [26].

In our study, we did not find a significantly different rate of negative appendectomies between the two groups. The more frequent use of MRI might explain why the aforementioned diagnostic challenges did not result in more misdiagnosis in pregnant women. However, this finding is in contrast to the study by Vasileiou et al. [19] which described a 3-fold rate of negative appendectomies in pregnant women.

Regarding intraoperative characteristics and postoperative outcomes, we found a higher rate of perforated appendicitis in pregnant women (16% vs. 10%). Few studies address the question of whether perforation is more common in pregnancy [3, 4, 19, 27]. Results on this question are conflicting, with some studies describing a higher rate of perforation [19, 27] while others do not confirm these findings [4]. In our study, atypical presentation did not lead to a longer time interval between admission and surgery, thus not explaining the higher rate of perforation.

Consistent with other studies, we found a longer postoperative hospital stay during pregnancy [19, 27]. Operating time was also longer, and we found a higher rate of complications measured using the Comprehensive Complication Index (CCI^®^), all of which were mild. Besides two cesarean sections which had to be performed within 30 days after appendectomy no major complications Clavien-Dindo ≥ 3 occurred among pregnant women.

Our study has strengths and limitations. It is one of the largest cohorts of pregnant women with acute appendicitis in the literature. We obtained data from initial examinations and provide detailed clinical data on the frequency of signs and symptoms as well as physical, laboratory and imaging findings. Due to its retrospective design, limitations include incomplete documentation and the risk of interpretation bias or inconsistency in the assessment of data. The time frame of the study spanned 15 years, so we cannot rule out some variability in clinical management of these patients. Diagnostic algorithms for appendicitis vary considerably from hospital to hospital and country to country, particularly with regard to the use of ultrasound and scoring systems. Nevertheless, we provide insights into the clinical presentation, management and outcome of a large number of women with acute appendicitis.

## Conclusion

The diagnosis of acute appendicitis during pregnancy presents a challenge to the clinician. Our results confirm that the clinical presentation differs between pregnant and non-pregnant women. In addition, ultrasound showed less diagnostic accuracy in pregnant women in our study. MRI is a useful tool to reduce uncertainty and the rate of negative appendectomies. Furthermore, our results underline that laparoscopic appendectomy during pregnancy is safe and does not increase the risk of serious complications.

## Data Availability

The original dataset generated during the current study is available from the corresponding author on reasonable request.
